# C-11, a New Antiepileptic Drug Candidate: Evaluation of the Physicochemical Properties and Impact on the Protective Action of Selected Antiepileptic Drugs in the Mouse Maximal Electroshock-Induced Seizure Model

**DOI:** 10.3390/molecules26113144

**Published:** 2021-05-24

**Authors:** Mirosław Zagaja, Aleksandra Szewczyk, Joanna Szala-Rycaj, Grzegorz Raszewski, Magdalena Chrościńska-Krawczyk, Michał Abram, Krzysztof Kamiński, Marta Andres-Mach

**Affiliations:** 1 Isobolographic Analysis Laboratory, Institute of Rural Health, Jaczewskiego 2, 20-090 Lublin, Poland; lasius1981@wp.pl (M.Z.); szewczyk.aleksandra@imw.lublin.pl (A.S.); szala-rycaj.joanna@imw.lublin.pl (J.S.-R.); 2 Department of Toxicology and Food Protection, Institute of Rural Health, Jaczewskiego 2, 20-090 Lublin, Poland; raszewskigj@gmail.com; 3 Department of Child Neurology, Medical University of Lublin, Chodzki 2, 20-093 Lublin, Poland; magdalena.chroscinska-krawczyk@umlub.pl; 4 Department of Medicinal Chemistry, Faculty of Pharmacy, Jagiellonian University Medical College, Medyczna 9, 30-688 Cracow, Poland; michal.abram@uj.edu.pl (M.A.); k.kaminski@uj.edu.pl (K.K.)

**Keywords:** antiepileptic drugs, maximal electroshock-induced seizures, pharmacokinetic/pharmacodynamic interaction, neuroprotection, physicochemical descriptors

## Abstract

C-11 is a hybrid compound derived from 2-(2,5-dioxopyrrolidin-1-yl) propanamide, with a wide spectrum of anticonvulsant activity and low neurotoxicity. The aim of this study was to determine the effects of C-11 on the protective action of various antiepileptic drugs (i.e., carbamazepine CBZ, lacosamide LCM, lamotrigine LTG, and valproate VPA) against maximal electroshock-induced seizures (MES) in mice, as well as its neuroprotective and physicochemical/pharmacokinetic properties. Results indicate that C-11 (30 mg/kg, i.p.) significantly enhanced the anticonvulsant action of LCM (*p* < 0.001) and VPA (*p* < 0.05) but not that of CBZ and LTG in the MES test. Neither C-11 (30 mg/kg) alone nor its combination with other anticonvulsant drugs (at their ED_50_ values from the MES test) affected motor coordination; skeletal muscular strength and long-term memory, as determined in the chimney; grip strength and passive avoidance tests, respectively. Pharmacokinetic characterization revealed that C-11 had no impact on total brain concentrations of LCM or VPA in mice. Qualitative analysis of neuroprotective properties of C-11, after a single administration of pilocarpine, revealed no protective effect of this substance in the tested animals. Determination of physicochemical descriptors showed that C-11 meets the drug-likeness requirements resulting from Lipinski and Veber’s rules and prediction of gastrointestinal absorption and brain penetration, which is extremely important for the CNS-active compounds.

## 1. Introduction

Epilepsy, one of the most common diseases of the nervous system, belongs to a group of social diseases. It is estimated that approximately 400,000 people suffer from this disease in Poland and approximately 60 million people worldwide, which constitutes about 1% of the human population [1]. Despite the availability of over 25 antiepileptic drugs (AEDs) worldwide, it is estimated that their effectiveness is in approximately 66% of all seizure patients, leaving 1/3 of the patients resistant to one of the available AEDs used in monotherapy [2,3]. The ineffectiveness of monotherapy with two consecutive drugs from the same group is the basis for the introduction of polytherapy with two or more AEDs, preferably with different mechanisms of action [4,5,6]. Such therapy, unfortunately, carries an increased risk of side effects.

Due to this, there is an urgent need to develop a new class of active compounds with anticonvulsant and neuroprotective properties while being non-toxic. Over the past few years, our team has focused on the search for new substances, both natural and synthetic, that have such properties, and may have a magnifying effect on commercially used AEDs, increasing their anticonvulsant activity [7,8,9,10,11,12,13,14].

**Figure 1:**
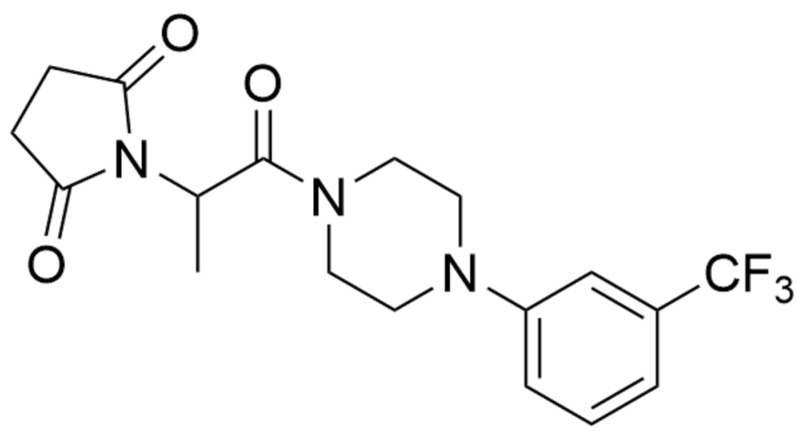
Structural formula of 2-(2,5-dioxopyrrolidin-1-yl) propanamide derivative (C-11).

Among a wide range of such substances is the 2-(2,5-dioxopyrrolidin-1-yl) propanamide derivative, C-11 (formerly KA-11) (Figure 1). This compound is a hybrid substance that was created as a result of the combination (hybridization) of fragments of the ethosuximide structure (pyrrolidine-2,5-dione derivative), levetiracetam LEV (butanamide derivative of pyrrolidin-2-one), and lacosamide LCM (compound with a benzylamide structure) [15]. Taking three AEDs with different modes of action into one substance may yield a compound with a multidirectional mechanism(s) of action, and as a result, broad.

Pharmacological studies conducted by our research team have revealed that the C-11 hybrid has a wide spectrum of anticonvulsant activity and is effective in three acute seizure models—MES, scPTZ, and 6 Hz (32 mA), after intraperitoneal administration in mice (Table 1). Additionally, C-11 effectively suppresses seizure progression in the kindling model of epilepsy caused by repeated injection of PTZ [16]. It should be emphasized that this substance combines protective properties of individual drugs forming a hybrid structure, which was observed in preclinical studies on animals. C-11 compound is more effective, and simultaneously, characterized by lower acute neurotoxicity than the commonly used valproic acid (VPA),which was assessed in the funnel test in mice (Table 1) [15]. Moreover, it appears that C-11 may positively influence epilepsy-induced depressive behaviors. This compound has also been shown to be effective in reducing pain responses in a tonic pain model and a chemotherapy-induced peripheral neuropathy model in mice [16].

We also evaluated C-11 influence on cognitive functions, neurodegeneration, and neurogenesis process in mice after chronical treatment in C57BL/6 mice. C-11 did not disturb the proliferation of newborn cells compared to the control mice and did not induce significant neurodegenerative changes in the mouse hippocampus. Behavioral studies did not indicate any disturbances in spatial learning and memory functions in the Morris Water Maze test after C-11 treatment [17]. In another experiment, we also assessed the impact of C-11 on neurogenesis and cognitive functions after pilocarpine (PILO)-induced Status Epilepticus (SE) in mice. The results obtained, where PILO SE mice were treated with C-11 and LEV, indicated markedly beneficial effects of C-11 on the improvement of neurogenesis compared to the PILO control and PILO LEV mice. Moreover, C-11 improved cognitive functions in PILO SE mice [18].

If an innovative substance that has a chemically different structure from the currently used AEDs exhibits anticonvulsant properties in experimental models of epilepsy, it is possible that such a substance could become a novel antiepileptic drug in the future. In general, AED candidates considered for preclinical evaluation are commonly assessed in combination with other, already established AEDs to confirm their effectiveness. Such a protocol is the same as the one in clinical trials, in which novel AEDs are usually co-administered with AEDs to provide the efficacy of novel antiepileptic drugs in patients with seizures [6,19].

Considering the above-mentioned facts, it seems interesting and necessary to continue experiments with C-11 in order to determine its anticonvulsant properties in combination with four various antiepileptic drugs (carbamazepine CBZ, lacosamide LCM, lamotrigine LTG, and valproate VPA) in the mouse maximal electroshock-induced seizure (MES) model, which is considered to be an experimental model of tonic–clonic seizure and, to a certain extent, of partial seizures with or without secondary generalization in humans [20]. That particular seizure model was chosen because of its impact on the evaluation of anticonvulsant properties of a variety of compounds and because of the possibility to determine their influence on commonly used drugs [20]. Additionally, to determine the acute adverse-effect profiles for the combinations of C-11 with CBZ, LCM, LTG, and VPA, three behavioral tests (chimney, passive avoidance, and grip-strength) were used. To confirm or exclude any pharmacokinetic background for the observed interactions between C-11 and the studied antiepileptic drugs, total brain concentrations of antiepileptic drugs were measured with HPLC techniques. Neuroprotective properties of C-11 were also assessed. For this purpose, experiments were conducted with the use of the neurodegenerative factor pilocarpine hydrochloride (PILO). Pilocarpine causes damage to neurons; therefore, it is commonly used to induce seizures and status epilepticus in animals [21,22,23,24,25]. Furthermore, using the online tool SwissAdme website, [26] the physicochemical properties of C-11 were determined.

## 2. Results

### 2.1. Effect of C-11 on the Anticonvulsant Activity of Various AEDs in the MES Model in Mice

**Figure 2:**
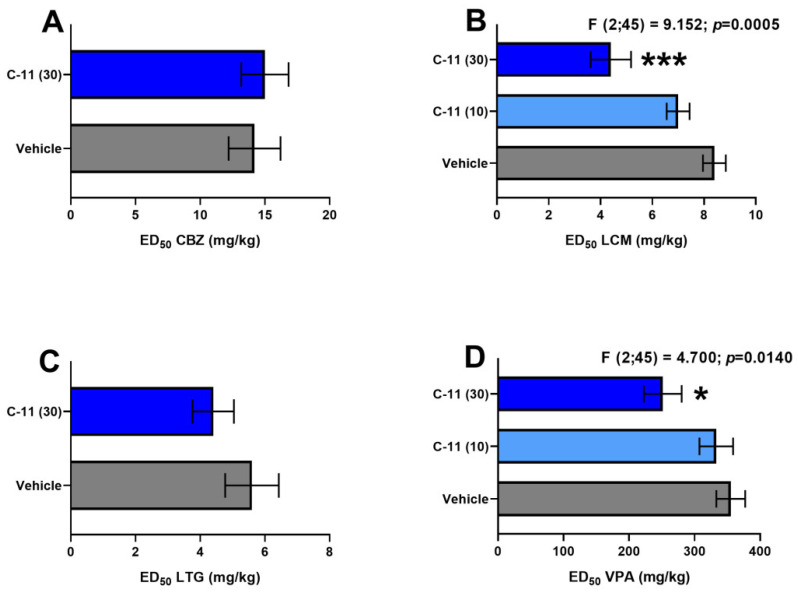
Effects of C-11 on the anticonvulsant potency of CBZ, LCM, LTG, and VPA in the MES model in mice. Columns represent median effective doses (ED_50_ in mg/kg ± SEM) of antiepileptic drugs (CBZ (**A**), LCM (**B**), LTG (**C**) and VPA (**D**)that protected half of the tested mice from tonic–clonic seizures. The log-probit method was used for calculating the ED_50_ values. *** *p* < 0.001, * *p* < 0.05 vs. control (LCM, VPA + vehicle-treated) animals (one-way ANOVA and post-hoc Tukey–Kramer test).

CBZ, LCM, LTG, and VPA when administered alone protected, in a dose-dependent manner, the animals from the tonic–clonic seizure model. Their ED_50_ values are presented in Figure 2A–D.

C-11 (30 mg/kg) co-administered with LCM significantly enhanced the anticonvulsant effect of the latter drug against maximal electroshock-induced seizures (F (2;45) = 9.152; *p* = 0.0005), by reducing its ED_50_ value from 8.4 mg/kg to 4.4 mg/kg (by 48%; *p* < 0.001) (Figure 2B). C-11 at a lower dose of 10 mg/kg did not significantly potentiate the anti-seizure activity of LCM in the MES test (Figure 2B).

In relation to the VPA, C-11 at 30 mg/kg markedly potentiated the anticonvulsant effects of this drug by decreasing its ED_50_ value from 355.2 to 251.5 mg/kg (by 29%; *p* < 0.05; Figure 2D). However, C-11 at a lower dose of 10 mg/kg had no significant effect on the antiepileptic properties of VPA in this experimental seizure model (Figure 2D).

In contrast, C-11 at doses of 30 mg/kg had no significant impact on the anticonvulsant action of CBZ and LTG in the MES test in mice (Figure 2A,C).

### 2.2. Effects of C-11 Alone and in Combination with Studied Aeds on Muscular Strength, Motor Coordination, and Long-Term Memory in Mice

C-11 administered alone at a dose of 30 mg/kg did not affect motor, skeletal muscular strength, and long-term memory in tested animals (Table 2). When C-11(30 mg/kg) was administered in combination with CBZ, LCM, LTG, and VPA at doses corresponding to their ED_50_ values from the MES test, long-term memory as determined in the passive avoidance test was unaffected (Table 2). Furthermore, none of the combinations studied impaired the skeletal muscular strength of the animals, as assessed by the grip-strength test (Table 2). Similarly, C-11 (50mg/kg) concomitantly administered with the AEDs had no significant impact on motor performance of the animals as assessed by the chimney test (Table 2). In regards to the AEDs administered alone at doses corresponding to their ED_50_ values from the MES test, the antiepileptic drugs had no significant impact on motor performance, skeletal muscular strength, and long-term memory in mice (Table 2).

### 2.3. Effect of C-11 on Total Brain AED Concentrations

**Figure 3:**
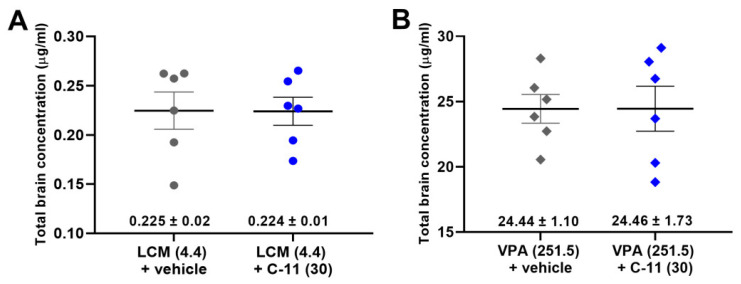
Influence of C-11 on total brain concentrations of LCM (**A**) and VPA (**B**) in mice. Scatter plots represent total brain concentrations of AEDs in µg/mL (as means ± SEM, as the error bars) (*n* = 6 mice/group). No statistical significance between the means were observed (unpaired Student’s *t* test).

Total brain concentrations of LCM and VPA for which ED_50_ values were significantly reduced by C-11 (30 mg/kg) administered alone did not differ from those determined for the combination of these drugs with C-11 (Figure 3A,B). Since C-11 at 30 mg/kg did not significantly affect the anticonvulsant potential of CBZ and LTG in the MES test, the total brain concentrations of this drug were not measured.

### 2.4. Influence of C-11 on Neuroprotection in Pilocarpine Convulsion in Mice

**Figure 4:**
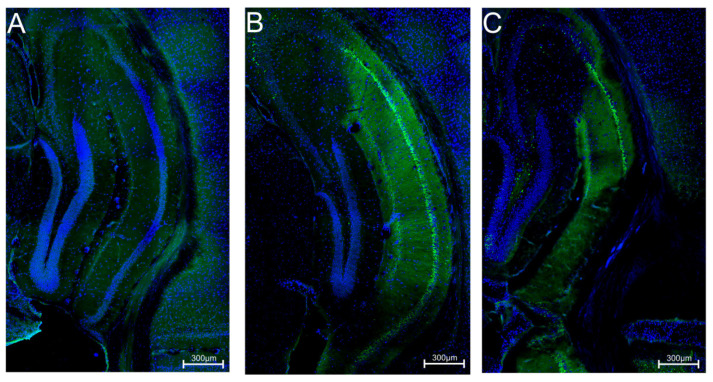
Qualitative assessment of neuroprotective properties of C-11. Results are presented in the form of pictures of hippocampal areas of selected hemisphere of one mouse from each test group. (**A**)—Control; (**B**)—PILO, 300 mg/kg; (**C**)—C-11, 100 mg/kg. The degenerate neurons are stained green; blue—cell nuclei.

Qualitative evaluation of potential neuroprotective properties of the C-11 compound administered at a dose of 100 mg/kg was carried out after a single administration of pilocarpine (PILO) at a dose of 300 mg/kg as a factor inducing permanent neuronal damage to the test groups. Results obtained from the FJB staining showed neurodegenerative changes for the C-11 group in the CA1–CA3 region of the hippocampus (Figure 4C), similar to the changes observed in the PILO control animals suggesting no neuroprotective effect of C-11 (Figure 4B). In contrast, no neurodegenerative changes were shown in the healthy control mice (Figure 4A).

According to the Racine scale, the PILO mice exhibited a high seizure score (4–5), characterized by generalized tonic, rearing, convulsion with status epilepticus (SE), and even death. The survival rate was 100% in the controls group. In the PILO and C-11 groups, the survival rate was similar (Table 3).

### 2.5. In Silico Physicochemical Descriptors Determination of C-11

The Lipinski and Veber’s rules are used to evaluate drug-like properties, which allow for determining whether a chemical compound has physicochemical properties that would make it suitable as an orally active drug in humans. The criteria of Lipinski’s rules are: molecular weight (MW) ≤ 500 Da, lipophilicity values (log *p*) ≤ 5, number of hydrogen bond donors (NHD) ≤ 5, and number of hydrogen bond acceptors (NHA) ≤ 10, and Veber’s rules include: rotatable bonds (NBR) ≤ 10 and polar surface area (PSA) ≤ 140 Ǻ2 [27,28] (Table 4).

The tested compound C-11complies with Lipinski’s and Veber’s rules. C-11 possesses a molecular weight below 500 Da, less than 5 hydrogen bond donors, less than 10 hydrogen bond acceptors, log *p* values < 5, number of rotating bonds (NBR) less than 10, and polar surface area (PSA) values lower than 140 Å2.

**Figure 5:**
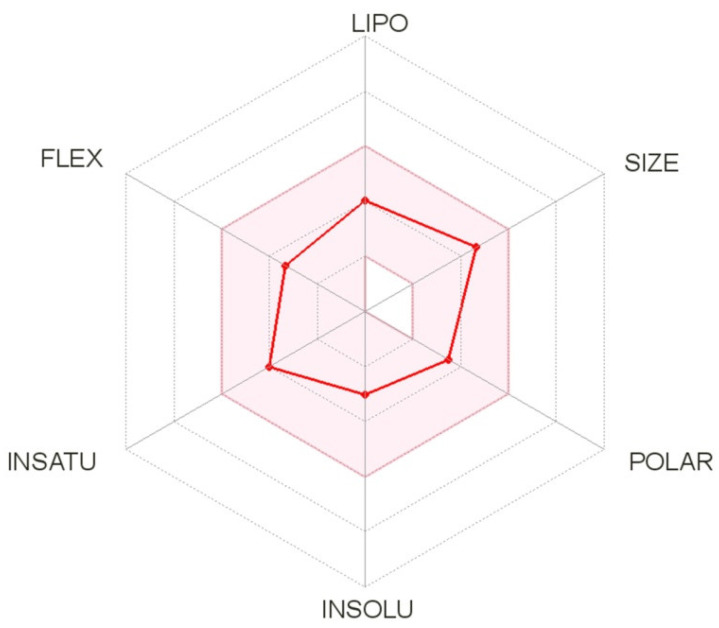
The bioavailability radar of compounds C-11 (LIPO—lipophilicity, POLAR—polarity, INSOLU—solubility, FLEX—flexibility, INSATU—saturation of the molecule).

The SwissADME website also provides radar charts that consider six physicochemical properties: lipophilicity, size, polarity, solubility, flexibility, and saturation of the molecule showing the relationship between chemical structures represented by given physicochemical descriptors and oral bioavailability. The physicochemical properties for C-11 are displayed as pink dots, while the pink area represents an acceptable range of physicochemical parameters according to Lipinski’s and Veber’s rules (Figure 5). It can be concluded that C-11 meets the drug-likeness requirements resulting from Lipinski’s and Veber’s rules.

**Figure 6:**
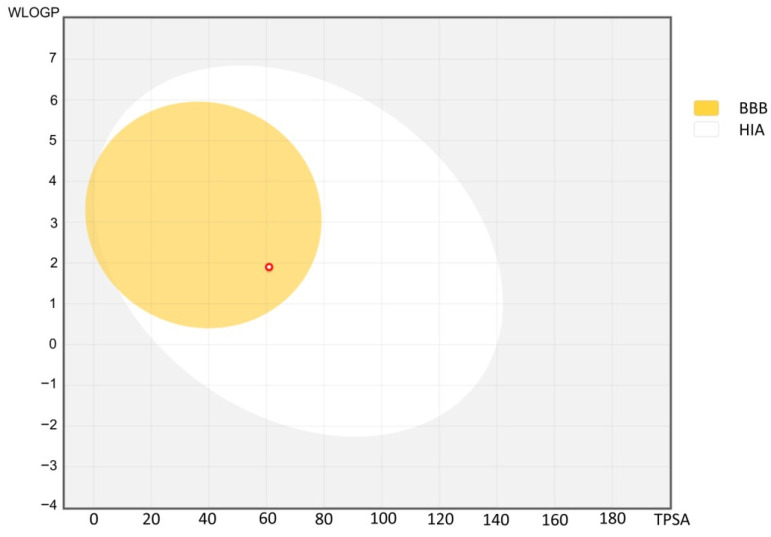
The BOILED-Egg predictive model for C-11 (visible as dot marked in red). The white region (HIA) is the physicochemical space of molecules with the highest probability of being absorbed from the gastrointestinal tract, and the yellow region (BBB) is the physicochemical space of molecules with the highest probability to permeate to the brain.

The Brain Or Intestinal Estimated permeation method (so called ‘BOILED-Egg’) is a predictive model that works by computing the lipophilicity and polarity of small molecules. Therefore, the SwissADME website provides ‘BOILED-Egg’ (Figure 6) showing the prediction of gastrointestinal absorption and brain penetration. In this predictive model, C-11 shows both high probability for good absorption from the gastrointestinal tract, and a high probability to cross the blood brain barrier.

## 3. Discussion

The aim of this study was to evaluate the effect of C-11 (pyrrolidine-2,5-dione derivatives) on the anticonvulsant action of four AEDs in the mouse model of tonic–clonic seizures. The use of a combination of C-11 and selected AEDs allowed to determine its impact on increasing the anticonvulsant effectiveness of the studied drugs. For combinations in which an increase in the protective effect was found, the type of interaction with the determination of whether the interaction was pharmacodynamic or pharmacokinetic was investigated.

We found that C-11 significantly enhanced the anticonvulsant action of LCM and VPA, but not that of CBZ and LTG in the MES test. To explain the nature of the pharmacodynamic interaction between C-11 and LCM or VPA, one should consider the fact that the mechanism of action of C-11 can be, at least in part, related to its ability to modulate the voltage-sensitive sodium and/or L-type calcium channels in neurons [15]. Kamiński et al. [15], using the [3H]BTX as radioligand, showed that C-11 was a relatively effective binder to the neuronal voltage-sensitive Na^+^ channel at the highest concentration (500 μM). The above results may suggest that the mechanism of anticonvulsant protection of C-11 is most probably related to its influence on the voltage-gated sodium channel. Moreover, in the binding assays for voltage-gated Ca^2+^ channels, C-11 exhibited stronger affinity to L-type Ca^2+^, even though it did not bind to the N-type Ca^2+^ channel. It should be emphasized that a modulation of neuronal L-type Ca^2+^ channel activity is an essential mechanism of action for topiramate (TPM), an AED with broad therapeutic spectrum [29,30]. Notably, C-11 proved to be a more effective binder to the voltage-gated L-type Ca^2+^ channel compared to the mentioned AED at a concentration of 100 μM.

LCM is a functionalized amino acid that produces activity in the MES test; therefore, its activity is considered to be similar to that of other AEDs through voltage-gated sodium channels [31,32]. However, LCM enhances the slow inactivation of voltage-gated sodium channels without affecting the fast inactivation of voltage-gated sodium channels, characteristic to many antiepileptic drugs, such as CBZ or LTG. Slow inactivation is similar to fast inactivation, but it does not produce complete blockade of voltage gated sodium channels, with both activation and inactivation occurring over hundreds of milliseconds or more. This activity causes LCM to only affect neurons that are depolarized or active for long periods of time, typical of neurons at the focus of epilepsy [15]. LCM administration results in the inhibition of repetitive neuronal firing, the stabilization of hyperexcitable neuronal membranes, and the reduction of long-term channel availability, but does not affect physiological function [33]. Moreover, it also modulates collapsin response mediator protein 2 (CRMP-2), preventing the formation of abnormal neuronal connections in the brain [34].

It seems that the increase in the anticonvulsant activities of LCM caused by C-11 in the mouse MES test may result from the fact that C-11 is a hybrid substance of which LCM is a part. However, the mechanisms of action of these drugs, as mentioned previously, are different, and moreover, the action of C-11 relies on the voltage-gated sodium channels based on their fast inactivation [16]. Presumably, having different mechanisms of action, C-11 and LCM act synergistically, potentiating the antiseizure effects of the two-drug mixture in the MES test. However, this needs experimental confirmation in further neurochemical studies.

VPA is another drug whose activity was enhanced by C-11 in the mouse tonic–clonic seizure model [35]. This drug constitutes an essential AED, without yet fully understood mechanism of activity, and because it is difficult to compare its mechanism to any specific one, it has been suggested that its therapeutic properties are a combination of numerous targets. Despite various reported pharmacologic effects, the antiseisure activity of VPA most likely results from the GABA mechanism. VPA increases the turnover of GABA, which might be connected to enhanced synaptic or extrasynaptic inhibition. At high concentrations, VPA was considered to affect voltage-gated sodium channels; however, contemporary research involving brain slice recordings did not provide a foundation for sodium channel block as an essential mechanism to support its clinical activity [36]. Likewise, there is little support to prove its effects on calcium channels. It is possible that that VPA possesses a pharmacologic action important for its antiseizure activity that remains uncovered [37].

It is highly likely that C-11, through the inhibition of voltage-gated sodium or calcium channels, contributes to the enhanced anticonvulsant potency of this drug. It is possible that the affinity of C-11 to both of the channels is higher than that of VPA and thus, C-11 potentiates its antiseizure action in the MES test. Although this explanation is highly speculative, it is very probable that C-11 enhances the blockade of sodium or calcium channels (or both of them) in neurons, contributing to the potentiation of the antiseizure effects of this drug, or as in the case of LCM, the mechanisms of action of C-11 and VPA are complementary. However, more advanced neurochemical and electrophysiological studies are required to elucidate this phenomenon.

On the other hand, C-11, as a sodium channel blocker, may compete with CBZ (AED with firmly established sodium channel blocker properties) in their affinity towards voltage-gated sodium channels. This could be the main reason whyC-11, when combined with CBZ, produced a barely additive interaction, even reducing the effect of this drug. It should also be emphasized that the inhibition of L-type neuronal calcium channels is the second important mechanism of CBZ activity [38]. Perhaps a similar situation occurs in the case of C-11 interactions with LTG which, apart from inhibitory action on sodium channels, may also block voltage- gated N- and P/Q-type calcium channels [39]. Moreover, LTG also demonstrates weak inhibitory effect on the serotonin 5-HT3 receptor, as well as weakly binds to other receptors including the Adenosine A1/A2, α1/α2/β adrenergic, dopamine D1/D2, GABA A/B, histamine H1, κ-opioid (KOR), mACh, and serotonin 5-HT2 [40]. Taking into account the multimodal mechanism of action of these two drugs, the lack of synergy between these AEDs and C-11 is perplexing. Perhaps in other experimental models of epilepsy, this compound would enhance the effects of these drugs; however, in order to confirm that, we need more preclinical studies.

Assessment of the adverse reaction profile in selected behavioral tests for CBZ, LCM, LTG, and VPA administered separately and in combination with C-11 (30 mg/kg) at doses corresponding to their ED_50_ values did not indicate any adverse effects of the tested combinations. None of the combinations tested significantly impaired motor coordination in the chimney test, skeletal muscle strength in the grip-strength test, and long-term memory in the passive avoidance test. Test results prove good tolerance of the combinations of tested drugs with C-11 among the tested experimental animals. Additionally, C-11 administered alone in a dose of 30 mg/kg does not have a negative impact on tested animals in all three behavioral tests, which is in line with previous studies where C-11 was non-toxic at doses even exceeding 1000 mg/kg of body weight of the tested animals in the chimney test [15]. Moreover, both the lower (50 mg/kg) and the higher doses of C-11 (100 and 150 mg/kg) did not cause disturbances in motor coordination in mice in the rotarod test at all speeds tested [15,16]. Socała et al. [16] confirmed that the C-11 compound administered at doses of 25 mg/kg and 50 mg/kg did not induce significant cognitive disorders in the passive avoidance test assessing the elements of long-term memory functioning in mice, while a high dose of C-11 (100 mg/kg) statistically significantly reduced the time of return to the shaded part of the room (the so-called latency time), which suggests that this compound, in high doses, may cause slight cognitive dysfunctions. In addition, Andres-Mach et al. [17] showed that C-11 at a dose of 20 mg/kg did not significantly impair the animal’s ability to orientate in space and the ability to learn and remember compared to the control group in the Morris Water Maze test;in the same study, 10 mg/kg LCM administered for 10 days caused significant dysfunctions in time, distance, and direct swim to the platform.

The results obtained from the assessment of the effect of C-11 on total LCM or VPA concentrations in brain tissue did not show a statistically significant increase in LCM or VPA in combination with C-11, which indicates the pharmacodynamic nature of the interaction between the tested substances.

Regarding the metabolism of LCM, this drug is removed from the organism via dual pathways: renal elimination of unchanged drug and metabolic degradation of the drug, thus using approximately 40% of the dose as unchanged active drug in the urine. In turn, about 60% of the dose is subject to metabolic degradation in two phases: the main phase 1 degradation processes are demethylation, deacetylation, and hydroxylation; the minor phase 2 metabolism contributes to glucuronidation. LCM is metabolized by several cytochrome P450 (CYP) enzymes (CYP2C19, CYP2C9, and CYP3A4) and CYP-independent mechanisms [41,42].

In the case of VPA, this drug is mainly metabolized in the liver. It is distinguished by glucuronidation, β oxidation in the mitochondria (both considered major routes accounting for 50% and 40% of dose, respectively), and cytochrome P450 (CYP)-mediated oxidation (considered a minor route, ~10%) [43,44]. VPA is known to be metabolized by the CYP enzymes: CYP2C9, CYP2A6, and to a lesser extent by CYP2B6 [44].

In a research conducted by Kamiński et al. [15] on the influence of C-11 on CYP activity, it has been stated that even with the highest applied doses of 10 and 25 µM, this compound exhibits a slight inhibitory effect on cytochrome CYP3A4 activity, which is responsible for the metabolism of over 50% of drugs [15]. Moreover, C-11 does not affect the function of CYP2D6, which is considered to be the second most important isoform of cytochrome P450 for possible metabolic interactions [16].

The fact that these enzymes are not affected by C-11 is likely to be caused by the nature of the interaction between this compound and test drugs, since these drugs, like many others, are metabolism-based and are mediated primarily via the microsomal CYP family of enzymes. As previously mentioned, among CYP isoforms, the CYP3A4 is responsible for the metabolism of more than 50% of medicines and the associated drug−drug interactions. The inhibition of these enzymes may decrease the metabolic clearance of a coadministered drug, resulting in elevated blood concentration, which may cause adverse drug effects or toxicity. This is crucial at an earlier stage of drug development to avoid the development of compounds with the potential to yield adverse drug interactions. Additionally, the determination of substance influence on the function of cytochrome P450 (CYP) is one of the most essential factors in the development of new drugs [45].

A preliminary, qualitative analysis of the potential neuroprotective properties of C-11 after a single administration of PILO showed no protective effect of this substance in the tested animals. The use of PILO as a neurodegenerative factor in the present study enabled the observation and the analysis of the degree of nerve cell degeneration, which was the basis for a preliminary assessment of neuroprotective properties of the test substance.

As it is well known that epilepsy causes the degeneration and death of neurons, neuroprotection appears to play a key role in monitoring this disease. All available LPPs can be divided into two groups—one characterized by a neuroprotective effect (benzodiazepines, LTG, LEV, PB, TPM, VPA, VGB), and the other without such potential, such as CBZ or PHT [46,47].

Among other AEDs, neuroprotective properties have been demonstrated for LCM in a gerbil cerebral ischemia model [48]. The results of the conducted studies showed that pre- and postoperative treatment of gerbils with LCM (25 mg/kg) had a protective effect on CA1 neuronal pyramidal cells in the hippocampus of tested animals. A study by Nirwan et al. [49] showed that LCM at the doses of 20 mg/kg and 40 mg/kg protected against PILO-induced status epilepticus in C57BL/6 mice, while preventing neurodegeneration and spatial memory impairment. Moreover, a number of in vitro studies proved that VPA protects neurons from glutamate-induced excitotoxicity [50], damage due to oxygen and glucose deprivation [51], as well as from oxidative stress [52]. In addition, in vivo studies showed that VPA protects neurons exposed to ischemic stroke [53].

Andres-Mach et al. [18] examined the neuroprotective properties of C-11 in human neurons and rat astrocytes under trophic stress and excitotoxicity conditions using the MTT test. The results proved that C-11, also in in vitro conditions, did not protect neurons; however, the results regarding the impact of C-11 on the nerve cell viability under trophic stress conditions in astroglia cell culture indicated that C-11 significantly induced the astrocytes viability. Furthermore, C-11 also effectively increased the number of astrocytes in the standard conditions (complete medium with a standard amount of trophic agents). The obtained data may suggest stimulating properties of C-11 on the astrocytes’ viability, as well as the nutritional effect on astrocytes under trophic stress conditions. This may be tied to the beneficial impact of C-11 on the secretion of trophic factors by astrocytes [54]. Taking into consideration the fact that neurotrophin production by astrocytes in response to brain tissue injury is a well-described mechanism of neuroprotection, such properties of C-11 are possible [55]. It seems that additional in vivo as well as in vitro tests are necessary to confirm or exclude the neuroprotective properties of this substance.

In summary, it can be concluded that C-11 seems to be a very interesting substance that increases the effect of LCM and VPA in the MES test; simultaneously, it does not affect their metabolism and does not cause behavioral disturbances in the tested animals when used in combination. In the future, additional preclinical studies are needed to confirm whether it is a good candidate for possible clinical trials, especially since it does not increase the effect of CBZ and LTG; it is likely that this susceptibility will not be active in CBZ- or LTG-resistant epilepsies.

Apart from low efficacy and high toxicity, the poor pharmacokinetics and bioavailability are the reasons for many new drug development failures. Therefore, the evaluation of two pharmacokinetic behaviors, namely gastrointestinal absorption and brain access, are crucial at very early stages of the drug discovery processes. Our physicochemical analyzes showed that C-11 exhibited the prediction of gastrointestinal absorption and brain penetration, which is extremely important for CNS-active compounds.

It should be emphasized that, in the development of novel antiseizure drugs, repeatability of research seems to be one of the basic criteria for introducing new drugs to common use. Improving the reproducibility of preclinical study results is one of the key elements in the development of new therapeutic agents [56]. One of the methods of solving this problem is defining common methods, terms, and units for data that are usually of interest for comparisons between various laboratories. Such an attempt was made by the International League Against Epilepsy and American Epilepsy Society through the introduction of the Common Data Elements (CDE) in preclinical epilepsy research. The use of CDEs is intended to increase rigor, standardization, and transparency of these researches [57,58] 

Confirmed from our recent studies, reliable protective index values for C-11 made it possible to classify this particular substance as a promising drug candidate for further clinical research. While some scientists argue that only compounds with protective index values over 5 in MES should be considered for further studies, others establish this limit at 2. In the second case, argumentation is based on the fact that the most efficacious AED –VAP has a protective index value of ~1.4. If more rigorous criteria had been applied in this case, VAP would never be available for patients. Since the protective index values for C-11 range between 9 and 17 in the MES test, this compound seems to be a promising drug candidate and should be eligible for further preclinical studies [15,59]. 

## 4. Materials and Methods

### 4.1. Animals

All study experiments were carried out on adult female Swiss mice weighing 20–25 g. The animals were kept in colony cages under standardized laboratory conditions: natural light–dark cycle 12/12 h, temperature 20–24 °C, air humidity 45–65%, and free access to tap water and food (chow pellets). After 7 days of adaptation to laboratory conditions, the animals were randomly assigned to experimental groups consisting of 8 mice. Each mouse was used only once and all tests were performed between 08:00 and 15:00 h. All the investigations were approved by the Local Ethical Committee at University of Life Sciences in Lublin (32/2019, 71/2020 and 6/2021) and were conducted in accordance with EU Directive 2010/63/EU for animal experiments as well as ARRIVE guidelines.

### 4.2. Drugs

The following drugs were used: pyrrolidine-2,5-dione derivativesC-11(Figure 1), carbamazepine CBZ (Polpharma, Starogard Gdanski, Poland), lacosamide LCM (Vimpat^®^, UCB Pharma, Brussels, Belgium), lamotrigine LTG (Lamictal^®^, GlaxoWellcome, Greenford, Middlesex, UK), valproate VPA (both from Sigma-Aldrich, Poznan, Poland), pilocarpine PILO (MP Biomedicals, LLC, Illkirch-Graffenstaden, France), and methyl scopolamine (Sigma-Aldrich, Saint Louis, MO, USA). The compound C-11 was obtained from the Department of Medicinal Chemistry, Jagiellonian University Medical College (Krakow, Poland) according to the procedure described previously [15]. All substances were suspended in a 1% solution of Tween 80 (Sigma-Aldrich, Saint Louis, MO, USA).

The studied drugs were administered intraperitoneally (i.p.) as follows: LTG—60 min, C-11, CBZ, LCM, and VPA—30 min, before electroconvulsion, motor coordination, grip-strength, and long-term memory tests brain sampling for the measurement of antiepileptic drug concentrations. C-11 and methyl scopolamine were administered intraperitoneally (i.p.) 30 min before pilocarpine-induced convulsion.

The pretreatment times before testing of the antiepileptic drugs were based on information about their biological activity from the literature [20], and our previous experiments [12,13,14]. The pretreatment time (30 min) before testing C-11 was established in our previous study as the time to peak of maximum anticonvulsant activity of C-11 [15].

Allsubstances were suspended in a 1% solution of Tween 80 (Sigma-Aldrich, Saint Louis, MO, USA) in water for injections (Baxter, Warszawa, Poland). All drugs were injected intraperitoneally (i.p.) with 1 mL syringes as a single injection, in a volume of 10 mL/kg.In the present study, CBZ was administered at doses ranging between 10 and 18 mg/kg, LCM at doses ranging between 3 and 10 mg/kg, LTG at doses ranging between 2 and 8 mg/kg, and VPA at doses ranging between 200 and 400 mg/kg.

### 4.3. Maximal Electroshock Seizure Test

Electroconvulsions were evoked by an electric stimulus (an alternating current 25 mA, 50 Hz,500 V, 0.2 s) generated by a rodent shocker (Hugo Sachs Elektronik, Freiburg, Germany) and delivered via ear-clip electrodes. Tonic hindlimb extension (i.e., hindlimbs of animals outstretched 180 to plane of the body axis) was established as the endpoint. ED_50_ is a median effective dose of the tested drug that protects 50% of mice against maximal electroshock-induced seizures. A dose–response curve was calculated on the basis of the percentage of mice protected according to Litchfield and Wilcoxon [60]. This experimental procedure has been described in detail in our previous studies [12,13,14].

C-11 was administered in doses that, per se, had no effect on seizure threshold in the maximal electroshock seizure threshold test. C-11 doses were selected based on previous studies where C-11 administered at a dose below 50 mg/kg protected mice from tonic hind limb extension after stimulation in MES test [15]. In addition, for ethical reasons, in accordance with the 3Rs rule, the maximal electroshock seizure threshold test (which would require at least additional 128 mice) was not performed.

### 4.4. Behavioral Tests

#### 4.4.1. Chimney Test

The effects of C-11 administered alone, AEDs administered alone, and their combinations (in doses reflecting their ED_50_ values from the MES test) on motor coordination in mice were determined with the chimney test, as described elsewhere [12,14,61].

#### 4.4.2. Grip-Strength Test

The effects of C-11 administered alone, AEDs administered alone, and their combinations (in doses reflecting their ED_50_ values from the MES test) on muscular strength of forelegs in mice were determined with the grip-strength test, as described elsewhere [12,14].

#### 4.4.3. Passive Avoidance Task

The effects of C-11 administered alone, AEDs administered alone, and their combinations (in doses reflecting their ED_50_ values from the MES test) on long-term memory (acquisition, learning, and remembering) in mice were determined with passive avoidance task, as described in details elsewhere [12,14,62].

### 4.5. Measurement of Total Brain Antiepileptic Drug Concentrations

The measurement of total brain concentrations of antiepileptic drugs was undertaken at the doses which correspond to their ED_50_ values from the MES test. Mice were killed by decapitation at times corresponding to the peak of maximum anticonvulsant effects for the antiepileptic drugs in the MES test. The whole brains of mice were removed from skulls, weighed, and homogenized using Abbott buffer (1:2 *w*/*v*) in an Ultra-Turrax T8 homogenizer (IKA Werke, Staufen, Germany). The homogenates were then centrifuged at 10,000 g for 10 min and the supernatant samples of 200 μL were collected.

The concentrations of LCM and VPA in brain homogenates were determined by a Dionex HPLC system (Dionex, Sunnyvale, CA, USA) with a UVD340S diode array UV detector, gradient pump P580 LPG, and manual injector (7725i Rheodyne) with a 20-µL loop. Chromatographic separation of LCM was performed ona ODS-2 C18 Hypersil (150 × 4.6 mm) column (Thermo Scientific, Darmstadt, Germany) packed with 5-µm particles using the mobil phase consisting of 0.05 M triethylammonium phosphate buffer solution–acetonitrile (70:30, *v*/*v*; pH −3.2) at ambient temperature. The flow-rate was 1.2 mL/min. For VPA, samples were injected into a ZORBAX SB-C18 (5 µm, 150 × 4.6 mm) column (Thermo Scientific, Darmstadt, Germany). Chromatography was performed using the mobil phase consisting of acetonitrile-phosphate buffer (50 mM; 45:55 *v*/*v*; pH 3.0), at ambient temperature. The flow-rate was 1.0 mL/min. The column eluates were monitored at 215 nm (LCM) and 254 nm (VPA) with a sensitivity of 0.01 absorbance units full scale.

Total brain concentrations of LCM and VPA are expressed in μg/g of wet brain tissue as means ± standard error (S.E.M.) of at least 6 separate brain preparations.

### 4.6. Neuroprotection of C-11

#### 4.6.1. Pilocarpine-Induced Convulsion in Mice

At the peak of C-11 activity (30 min, dose100 mg/kg) experimental animals were injected i.p. with a single dose of PILO 300 mg/kg; 30 min prior to injection of PILO, mice were given methyl scopolamine (1 mg/kg; i.p.) to reduce the peripheral cholinergic effects of PILO.

Control mice were age-matched with treated mice and administered a comparable volume of vehicle after the initial methyl scopolamine treatment. The mice were observed continuously for 60 min for any behavior indicative of seizures, and graded according to a modified version of the Racine scale [63]. Status Epilepticus (SE) incidence, mortality rate, and convulsion onset time were also recorded. Convulsion was defined as the occurrence of grade 4–5 seizures based on the Racine scale. When mice experienced grade 4–5 seizures or SE for 60 min, the convulsions were terminated by an intraperitoneal injection of diazepam (1 mg/kg) to reduce mortality.

#### 4.6.2. Brain Slice Preparation

At 72 h after treatment, mice were anesthetized with isoflurane anesthesia with premedication of analgesic drug, and perfused with ice-cold saline, followed by freshly prepared, ice-cold 4% paraformaldehyde. The brains were removed, processed, and coronal sections were cut on a vibratome (Leica VT1000 S, Wetzlar, Germany) at a thickness of 40 μm [17].

#### 4.6.3. Fluoro-Jade B Staining

To identify neurons undergoing degeneration in mice brain slices, Fluoro-Jade B (FJB) staining was used, as an established detection technique for degenerating neurons described by [64]. To properly recognize neurodegenerative changes, as well as to make sure that FJB staining worked correctly, we used PILO SE brain slices as a positive control of neuronal damage. The stained slices were photographed using a Nikon A1R confocal system microscope (Tokyo, Japan).

### 4.7. In Silico Physicochemical Descriptors Determination

Physicochemical properties of C-11 and BOILED-Egg predictive model were determined using the online tool–SwissADME website [26].

### 4.8. Statistics

The ED_50_ values with their respective 95% confidence limits were calculated in the computer log-probit analysis according to Litchfield and Wilcoxon (1949). Then, the standard errors (SEMs) of the mean values were assessed on the basis of confidence limits. Multiple comparisons of the ED_50_ values (±SEM) from the MES test were performed using one-way analysis of variance (ANOVA) followed by the post-hoc Tukey/Kramer test.

Qualitative variables from the chimney test were compared using the Fisher’s exact probability test. The results from the grip-strength test were verified with one-way ANOVA, followed by the post-hoc Bonferroni’s test. The results obtained in the step-through passive avoidance task were statistically evaluated using Kruskal–Wallis nonparametric ANOVA, followed by the post-hoc Dunn’s test.

Total brain antiepileptic drug concentrations were statistically compared using the unpaired Student’s t-test. Differences among values were considered statistically significant if *p* < 0.05.

## 5. Conclusions

Based on the results from this study, one can ascertain that C-11 pharmacodynamically potentiates the anticonvulsant action of LCM and VPA with no adverse effects among the tested drugs. C-11 had no significant impact on the protective action of CBZ and LTG against MES test in mice, indicating neutral interaction between these drugs. The combination of C-11 with CBZ and LTG is neutral from a preclinical point of view, because C-11 did not enhance the anticonvulsant potency of these drugs in experimental animals in the MES test. C-11, after a single administration of pilocarpine, revealed no neuroprotective effect in the tested animals. Physicochemical descriptors determination revealed that C-11 has good drug-likeness parameters as well as high probability for good absorption from the gastrointestinal tract, and a high probability to cross the blood brain barrier. Taking into account our results, there is no doubt that modifications of the chemical structure of compounds and/or currently available antiepileptic drugs might contribute to the development of new drugs, more favorable and better tolerated than conventional antiepileptic drugs.

## Figures and Tables

**Table 1:** Antiseizure and acute adverse effects of C-11 in the three seizure models and chimney test in mice.

Pretreatment Time (min)	ED_50_ MES (mg/kg)	ED_50_ PTZ (mg/kg)	ED_50_ 6Hz (mg/kg)	TD_50_ (mg/kg)	PI
30	88.4 ± 8.5	59.9 ± 4.0	21.0 ± 6.6	>1500	>16.97 (MES) >25.04 (PTZ) >71.43 (6 Hz)
60	85.1 ± 5.5	88.7 ± 3.1	35.0 ± 8.2	823.6 ± 107.9	9.68 (MES) 9.28 (PTZ) 23.53 (6 Hz)

Results are ED_50_ (±S.E.M) and TD_50_ (±S.E.M) values of C-11 that protected 50% of the mice from MES, PTZ, 6Hz-induced seizures, and impaired motor coordination in 50% of mice challenged with chimney test, respectively [15].

**Table 2:** Effects of C-11 and its combinations with classical antiepileptic drugs on long-term memory, muscular strength, and motor performancein mice.

Treatment (mg/kg)	(1) Retention Time (s)	(2) Grip Strength (gf)	(3) Motor Coordination Impairment (%)
**Vehicle**	180 (173; 180)	98.51 ± 5.37	0
**C-11 (30)**	151 (25; 180)	94.89 ± 4.09	25
**VPA (251,5)**	137 (35; 180)	89.63 ± 6.15	0
**VPA (251,5)+ C-11 (30)**	156 (107; 180)	80.26 ± 4.94	12.5
**CBZ (15.0)**	180 (74; 180)	113.5 ± 4.25	0
**CBZ (15.0) + C-11 (30)**	180 (135; 180)	102.2 ± 4.73	0
**LCM (4.4)**	180 (130; 180)	94.80 ± 5.61	12.5
**LCM (4.4)+ C-11 (30)**	180 (29; 180)	98.26 ± 6.30	25
**LTG (4.4)**	180 (151; 180)	104.8 ± 6.41	0
**LTG (4.4) + C-11 (30)**	180 (161; 180)	97.94 ± 4.82	37.5

Results are presented as: (1) median retention times (in seconds; with 25th and 75th percentiles in parentheses) from the passive avoidance task, assessing long-term memory in mice; (2) mean grip-strengths (in Newtons ± S.E.M.) from the grip-strength test, assessing muscular strength in mice; and (3) percentage of animals showing motor coordination impairment in the chimney test in mice. Each experimental group consisted of eight mice. Statistical analysis of data from the passive avoidance task was performed with nonparametric Kruskal–Wallis ANOVA test, whereas those from the grip-strength test were analyzed with one-way ANOVA followed by Bonferroni’s post-hoc test. Fisher’s exact probability test was used to analyze the results from the chimney test. All drugs were administered i.p. at times scheduled from the maximal electroshock-induced seizures and at doses corresponding to their ED_50_ values against maximal electroconvulsions in mice.

**Table 3:** Effect of C-11 on pilocarpine (PILO)-induced convulsions and lethality. Data on survivors and the number of animals with status epilepticus (SE) calculated as percentages.

Groups	Number of Animals	Percentage Convulsion (%)	Percentage SE (%)	Percentage of Survival (%)
Control	10	0	0	0
PILO	10	100	100	50 (5/10)
C-11	10	100	100	60 (6/10)

**Table 4:** Drug-likeness parameters estimated according to Lipinski and Veber rules.

Compound	Lipinski Rule				Veber Rule	
	MW ≤500	LogP ≤5	NHD ^a^ ≤5	NHA ^b^ ≤10	NBR ^c^ ≤10	TPS ^d^ ≤140
**C-11**	383.37	1.98	0	6	5	60.93

^a^ NHD: number of hydrogen bond donors; ^b^ NHA: number of hydrogen bond acceptors; ^c^ NBR: number of rotatable bonds; ^d^ TPSA: total polar surface area.

## Data Availability

The data supporting reported results can be found in the laboratory databases of Institute of Rural Health.

## Sample Availability

Samples of the compounds studied in the present work are available from the authors at reasonable request.
